# Basal Xenobot transcriptomics reveals changes and novel control modality in cells freed from organismal influence

**DOI:** 10.1038/s42003-025-08086-9

**Published:** 2025-04-22

**Authors:** Vaibhav P. Pai, Léo Pio-Lopez, Megan M. Sperry, Patrick Erickson, Parande Tayyebi, Michael Levin

**Affiliations:** 1 Allen Discovery Center at Tufts University, Medford, MA USA; 2 Wyss Institute for Biologically Inspired Engineering, Harvard University, Boston, MA USA

**Keywords:** Biological techniques, Biophysics, Biotechnology, Systems biology

## Abstract

Would transcriptomes change if cell collectives acquired a novel morphogenetic and behavioral phenotype in the absence of genomic editing, transgenes, heterologous materials, or drugs? We investigate the effects of morphology and nascent emergent life history on gene expression in the basal (no engineering, no sculpting) form of Xenobots —autonomously motile constructs derived from Xenopus embryo ectodermal cell explants. To investigate gene expression differences between cells in the context of an embryo with those that have been freed from instructive signals and acquired novel lived experiences, we compare transcriptomes of these basal Xenobots with age-matched Xenopus embryos. Basal Xenobots show significantly larger inter-individual gene variability than age-matched embryos, suggesting increased exploration of the transcriptional space. We identify at least 537 (non-epidermal) transcripts uniquely upregulated in these Xenobots. Phylostratigraphy shows a majority of transcriptomic shifts in the basal Xenobots towards evolutionarily ancient transcripts. Pathway analyses indicate transcriptomic shifts in the categories of motility machinery, multicellularity, stress and immune response, metabolism, thanatotranscriptome, and sensory perception of sound and mechanical stimuli. We experimentally confirm that basal Xenobots respond to acoustic stimuli via changes in behavior. Together, these data may have implications for evolution, biomedicine, and synthetic morphoengineering.

**Figure:**
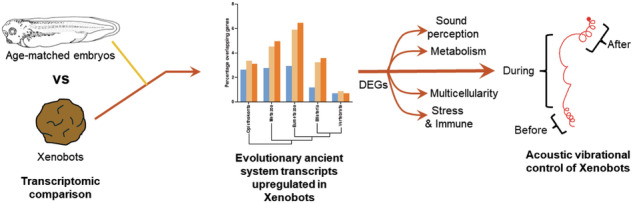

## Abstract (web summary)

Basal Xenobot transcriptome shows unique up-regulation of evolutionarily ancient systems driven by its nascent emergent life history, including changes of metabolism and functional sensory perception of acoustic vibration.

## Introduction

The standard research paradigm of developmental and synthetic biology seeks to discover how gene expression drives specific anatomical and behavioral outcomes^1–6^. However, it is also well-understood that transcriptional machinery itself is sensitive to external cues^7–13^. Rapid changes of gene expression can be induced by exposures to genetic, biochemical, biomechanical, bioelectrical, and materials-mediated influences. On a much longer timescale, the properties of gene expression profiles in vivo are thought to be determined by evolutionary selection, optimizing fitness to specific environment, life history, and lived experiences^14–18^. Here, we sought novel aspects of the responsiveness of gene expression in a setting in which a non-canonical multicellular, functional form is achieved without transgenes, chemical signals, foreign nanomaterials, or other added influence.

A fascinating set of questions concerns the origins of species-specific transcriptomic profiles, normally shaped by eons of selection over the functionality of those forms in a specific environmental context. Unique aspects of these questions can be addressed in synthetic systems in which the entities have not had a history of selection in their current multicellular embodiment. Numerous synthetic life forms have recently been produced^19–29^; however, many questions remain to be answered via analysis of transcriptomic profiling in these synthetic living configurations. In this study, we utilize one such living system to understand how synthetic morphology might result in transcriptomic changes. Our goal was to characterize unique transcriptomic changes in this synthetic living system in comparison to its native (wild-type) embryo context and understand the effect of novel lived experiences (and removal of instructive endogenous signals) on the transcriptome.

Multiple different kinds of autonomously moving biobots^23,30,31^ can be derived from *Xenopus* embryonic cells. They offer self-organization (a kind of developmental morphogenesis), as they need no scaffold in order to form and mature into functional constructs. They can form from a single tissue (prospective skin/epidermis) or from a combination of multiple tissues (e.g., skin/epidermis and muscles), can be of multitude of shapes, and be actuated by either the muscle contraction or coordinated beating of cilia^32–35^. They show self-healing and emergent group behaviors including kinematic self-replication^33,34^. This overall class is referred to as “Xenobots”^32^ to emphasize their utility as potentially programmable living materials—a model system in which to learn to control the plasticity of living active matter toward applications that shed light on life-as-it-can-be^36–42^. In this study, we use the *basal* (spherical) Xenobots^32^ derived from completely wild-type ectodermal explant cells with no drugs, synthetic biology circuits, nanomaterials, sculpting, or anything else added. Hence, we can observe latent behaviors of these cellular collectives that are released by lifting signaling constraints present when they were part of the larger organism^43–48^.

The basal Xenobots are autonomously motile, self-assembling constructs derived from frog (*Xenopus laevis*) embryonic ectodermal explants (colloquially referred to in developmental biology studies as “animal caps”) as starting material^33^. The *Xenopus* ectodermal explants (animal caps) have been studied for several decades and serve as an excellent model for studying epidermal cell fate and patterning, as well as testing the plasticity and responsiveness of these cells to various inducers and inhibitors^49–55^. Also, mucociliary organoids developed from these *Xenopus* ectodermal explants have served as excellent models for understanding dynamics of mucociliary epidermis formation and maintenance^56–61^. However, we cultured these ectodermal explants until they formed *autonomously-moving* atypical entities, aka the basal Xenobots used in this study. We were particularly interested in studying the transcriptomic changes in these autonomously-moving Xenobots. Recently, elegant temporal transcriptomic analysis has shown epidermal cell fate specification during the transformation of *Xenopus* ectodermal explants (animal caps) to autonomously-moving atypical entities (the basal Xenobots used in this study)^56,62^. However, in this study, we sought to broadly analyze genome-wide transcriptomic changes well beyond epidermal cell fate specification in response to a not previously selected-for embodiment and nascent life history of these autonomously-moving atypical entities—as system that self-assembles without being forced onto an engineered scaffold and exhibits autonomous motion behavior (being functionally closer to a proto-organism than a typical organoid). We see these autonomously-moving atypical entities (basal Xenobots) as an important biorobotics platform because, once their properties are understood, they can be used as a testbed in which to improve our understanding and ability to control growth and form, and ultimately deploy them for many useful purposes. Unlike conventional robotic materials, living cells offer numerous complex behaviors and responses; understanding the plasticity and molecular-biological responses, of cell collective entities in new contexts is a critical part of morphogenic engineering efforts^19,30,31,63–65^.

To advance this engineering roadmap, as well as more broadly understand how transcriptomes respond to internal and external environment changes (e.g., for biomedical purposes, as well as basic evolutionary developmental biology), we compared the transcriptome of basal Xenobots to those of age-matched embryo controls. Our goal was to characterize transcriptomic changes in cells released from the constraints and influences of the rest of the embryo, and thus following a different developmental trajectory and nascent emergent life history. Importantly, we did not focus on gene expression missing from Xenobots, because it was expected that, being made of just one tissue type, they would lack many transcripts that normal embryos expressed. Instead, we asked what transcripts, other than epidermal markers (expected to be enriched), Xenobots expressed that their age-matched embryos did not. We found at least 537 transcripts to be uniquely upregulated in these Xenobots, representing functional classes such as locomotion apparatus assemblies (cilia and motor proteins), structure formation and multicellularity, stress and immune response, metabolism, and even sensory perception of sound and mechanical stimuli. Functional experiments confirmed that basal Xenobots, unlike age-matched *Xenopus* embryos, indeed respond to acoustic vibration stimuli by changing their motion behavior. Phylostratigraphic analysis shows that the majority of these transcriptomic shifts are towards evolutionary ancient transcripts and systems. These changes suggested that alteration of morphology and nascent emergent life history, in wild-type cells, were enough to strongly affect the transcriptome (morphology/behavior driving gene expression). Moreover, we found a significantly greater inter-individual gene variability in these Xenobots compared to their age-matched *Xenopus* embryos, suggesting exploratory adaptation in the transcriptional space to adapt and survive in their new embodiment^66–69^. Another unique aspect that these Xenobots allowed us to investigate was the nature of organismic death and its effects on gene expression. By making a Xenobot out of an embryonic source of cells, the original embryo “dies”—it is no more; but the cells persist and continue on in a new embodiment. Our analysis revealed enrichment of thanatotrascriptomic^70–74^ genes, seen previously in studies of human post-mortem molecular physiology. Taken together, these data shed light on the feedback of form and function onto cellular genetics, in the context of evolved and engineered living beings transitioning across levels of organization.

## Results

### RNA sequencing analysis shows no major transcriptional changes between stage 35/36 Xenopus embryos raised in 0.1X MMR and 0.75X MMR

One major difference in the rearing condition of *Xenopus* embryos and basal Xenobots used in this study is the MMR (Marc’s Modified Ringers) medium concentration: embryos are reared in 0.1X MMR, Xenobots are reared in 0.75X MMR (a higher concentration of salts). Thus, we first asked whether raising embryos in 0.75X MMR induces any significant transcriptional changes in comparison to embryos raised in 0.1X MMR. We performed RNA sequencing analysis on stage 35/36 *Xenopus* embryos (stage corresponds to the formation of mature autonomously-moving Xenobots on day 7 at 14 °C after explanting ectodermal explants (animal caps) from stage 9 embryos) raised in either 0.1X MMR or raised in 0.75X MMR after stage 9 (stage at which ectodermal explants are removed from embryos and placed in 0.75X MMR to make the basal Xenobots). We collected RNA from three replicate samples (each sample containing ten stage 35/36 embryos) for each condition (0.1X and 0.75X MMR) (Supplementary Fig. 1A). Overall, we observed no separation between groups by principal component analysis (Supplementary Fig. 1B). However, after surrogate variable analysis and batch-correction, we did observe some separation of the clusters (Supplementary Fig. 1C). We thus performed differential gene expression analysis and found that in the histograms of significance the *p* value histograms are relatively flat, with the proportions of the true null hypothesis (non-significant genes) to be 0.996 (Supplementary Fig. 1D) and all FDRs (False Discovery Rates) to be near one (Supplementary Fig. 1E). These results indicate minimal changes in the overall transcriptome of 0.75X MMR raised embryos versus 0.1X MMR raised embryos. Differential expression analysis showed that out of ~24,500 transcripts, only 6 genes were significantly changed (FDR < 0.05) with a log fold change > 2 in the 0.75X MMR raised embryos (Supplementary Fig. 1F and Supplementary Data 1). These results show that overall raising embryos in the higher salt medium at 0.75X MMR results in very little transcriptional change – the transcriptome is not highly sensitive to external conditions.

### RNA-sequencing analysis shows major transcriptional changes in the basal Xenobots compared to age-matched stage 35/36 Xenopus embryos

**Fig. 1:**
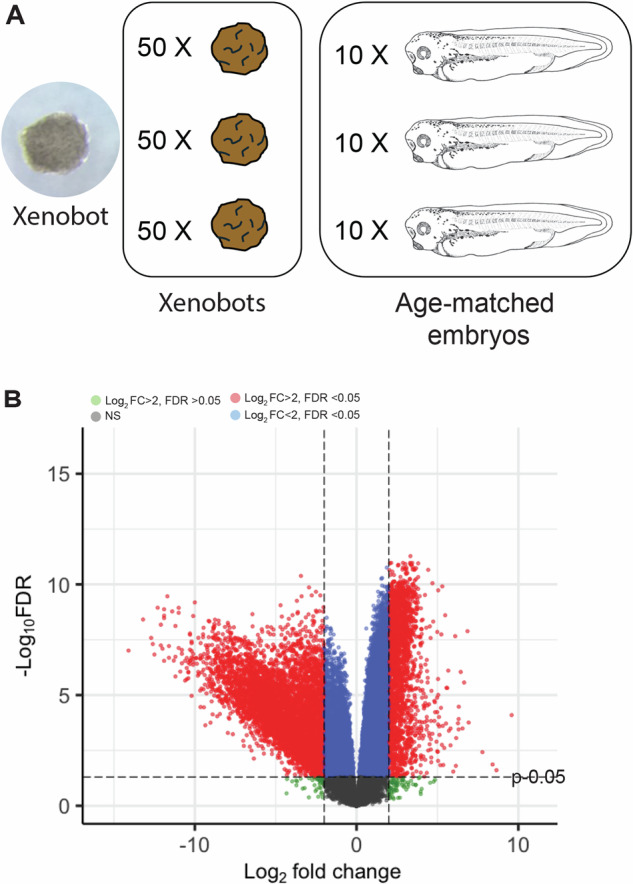
Major transcriptional changes in Xenobots compared to age-matched stage 35/36 *Xenopus* embryos. **A** A live Xenobot and schematic of the experimental setup with three replicates for each Xenobot pool and age-matched stage 35/36 Xenopus embryo pool with each replicate having fifty and ten samples, respectively. Illustration from Nieuwkoop and Faber^178^ (**B**) Volcano plot for differential expression between Xenobots and age-matched stage 35/36 Xenopus embryos. Significantly changed genes are highlighted in red.

The basal Xenobots are derived by explanting ectodermal explant (animal cap) tissue from stage 9 *Xenopus* embryos, and reach maturity by day 7: fully differentiated cells, peak cellular health, and peak robust autonomous movement behavior^33^. To test our hypothesis that these Xenobots would have unique and distinct transcriptomic changes compared to normal *Xenopus* embryos, we compared these Xenobots’ transcriptome to that of age-matched (stage 35/36) control *Xenopus* embryos that were reared in identical conditions (incubation temperature and time) as the Xenobots. We collected RNA from three pooled samples for each condition (Xenobots and age-matched stage 35/36 *Xenopus* embryos) (Fig. 1A) and performed RNA-seq to compare the two transcriptomes. Principal component analysis using surrogate variables and batch-correction showed marked separation between the clusters of Xenobot transcriptome and that of age-matched *Xenopus* embryos (Supplementary Fig. 2). Differential expression analysis showed that out of 28,009 transcripts, 1962 transcripts were significantly up-regulated (FDR < 0.05) with log fold change > 2 in these Xenobots compared to age-matched embryos and 5053 transcripts were significantly down-regulated (FDR < 0.05) with log fold change < −2 in these Xenobots compared to age-matched embryos (Fig. 1B). These results show that overall, these Xenobots have major transcriptional differences from age-matched *Xenopus* embryos.

### The basal Xenobots have greater inter-individual gene count variability than age-matched stage 35/36 Xenopus embryos

In the principal component analysis (Supplementary Fig. 2), the three Xenobots samples show much wider separation compared to the three age-matched embryo samples. Previous work has shown that cells experiencing novel stressors can randomly change their gene expression profiles until they find configurations that resolve the stress, and that individual cells discover different expression profiles that solve the same problem^66,68,69,75,76^. We hypothesized that the process of removal from the embryo serves as a novel stressor for the nascent Xenobot tissue, requiring the tissue to adapt to its new embodiment in ways that evolution did not prepare it for. In contrast, while embryos are indeed capable of solving novel problems to achieve their developmental goals^77^, their developmental trajectory is more stereotyped and likely requires less transcriptional exploration than do these Xenobots.

**Fig. 2:**
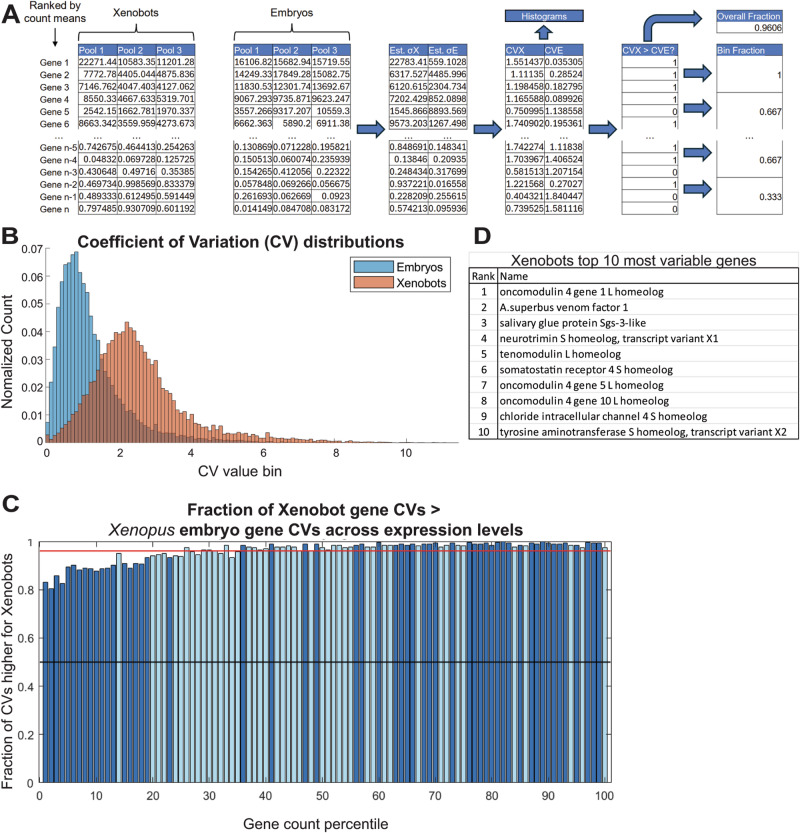
Inter-individual variation in gene counts is greater in Xenobots than in age-matched *Xenopus* embryos for most genes. **A** Schematic of the analysis method. All genes were ranked by their mean count value across all 6 data pools, after which the standard deviations of the gene counts amongst individual Xenobots and individual age-matched *Xenopus* embryos were estimated (Est. σX and Est. σE) and used to calculate the coefficients of variation (CVs) for each (CVX and CVE). **B** Histograms of the normalized distributions of CVs for Xenobots and age-matched embryos genes. Xenobots have significantly higher CVs and wider CVs distribution than age-matched embryos (Wilcoxon rank sum test, *p* = 0). Where the CVE distribution had a mean of 1.2537 and standard deviation of 0.9716, and the CVX distribution had a mean of 2.6115 and standard deviation of 1.3609. **C** Comparison of gene expression variation between Xenobots and age-matched embryos across expression levels. The genes for which CVX > CVE were noted, and of all the included genes, 96.06% had greater CVX than CVE. The gene list was then divided into 100 equal-size bins (percentiles), and the fraction of each bin for which CVX > CVE was plotted. Each bar represents one bin of genes ranked from lowest to highest gene counts, and its height indicates the fraction of genes in the bin for which CVX > CVE. The black line marks 0.5. For all bins, most genes in the bin had a greater CV for Xenobots than age-matched *Xenopus* embryos. The red line shows the overall fraction of genes for which CVX > CVE (0.9606), and the dark blue bins were found to be significantly different from this value (*p* < 0.05), while light blue bins were not (*p* > 0.05) according to a permutation test, indicating that the trend of increasing bin value with increasing gene count was not due to chance. **D** Table showing top 10 most variable genes in Xenobots.

We compared the variability of individual transcripts among biological samples using the method in Fig. 2A. Briefly, all 28,009 gene transcripts were ranked based on their mean count values across all samples (3 Xenobot samples and 3 *Xenopus* embryo samples), combined, and genes for which any sample had a value of 0 were removed. The standard deviations of individual gene transcripts among Xenobot samples and *Xenopus* embryo samples were estimated and used to calculate the coefficient of variation (CV) for each gene as plotted in Fig. 2B histograms. In Xenobots, genes have significantly higher inter-individual CVs and a broader range of CVs than embryos (Wilcoxon Rank Sum test, *p* = 0) (Fig. 2B). The ranked gene list was split into 100 equal-size bins (percentiles), and the fraction of genes in each bin for which CV of the Xenobots (CVX) was greater than the CV of the *Xenopus* embryos (CVE) was plotted (Fig. 2C). We found that almost all genes have a greater CV for Xenobots than *Xenopus* embryos (96.06% of all included genes, shown by the red line) (Fig. 2C). Also, the general trend of increasing bin fraction with higher gene count percentile indicated that, the higher the gene expression, the higher is the variability of that gene in Xenobots compared to *Xenopus* embryos (Fig. 2C). A permutation test (see “Methods”) revealed that these deviations from the expected (null hypothesis) bin fraction value (0.9606 the red line in Fig. 2C) across gene count percentiles were statistically significant (dark blue colored bins with *p* < 0.05). Among the top ten most variable genes in these Xenobots (Fig. 2D), *Oncomodulin* gene which encodes a protein with widely distinct functional roles^78^ is most prominent with three *oncomodulin* genes in the top ten (Fig. 2D). Other interesting genes include *A. superbus venom factor 1*, *salivary glue protein-3 like protein*, *neurotrimin, tenomodulin, somatostatin receptor 4, chloride intracellular channel 4* (Fig. 2D). Thus overall, there is significantly greater gene count variability in these Xenobots compared to age-matched *Xenopus* embryos, possibly indicating exploratory adaptation in the transcriptional space to adapt and survive in their new embodiment.

### Stringent curation of Xenobot transcripts

The basal Xenobots are derived from ectodermal explants (animal cap explants), which are primarily ectodermal progenitor cells. Most cells and tissues belonging to mesoderm and endoderm are absent in these Xenobots as compared to *Xenopus* embryos. Hence, mesoderm and endoderm transcripts would come out as highly downregulated in these Xenobots compared to age-matched embryos, largely due to absence of those cells and tissues in these Xenobots (false positives). Similar to previous studies^56^, we found no clear path to resolve and differentiate which downregulated transcripts (out of the 5053 downregulated transcripts) are changed due to absence of mesoderm and endoderm (false positives) and which transcripts are truly downregulated in these Xenobots compared to age-matched *Xenopus* embryos (true positives). Hence, we did not further analyze the downregulated transcripts dataset.

Conversely, Xenobots are fully derived from ectoderm, but ectoderm only forms a small portion of whole embryo (~10%). Thus, in a head-to-head comparison, ectodermal genes would be artificially enriched in these Xenobots, resulting in false positives due to simple proportional enrichment of ectodermal tissues. To adjust for this, we subtracted out the artificially enriched ectodermal/epidermal-specific transcripts from the 1962 upregulated transcripts to curate a list of transcripts uniquely upregulated in these Xenobots, which could not be explained by the increased prevalence of ectodermal cells. To achieve this, we used Klein tools^79^—a database of single-cell transcriptome of stage 22 entire *Xenopus* embryo. We subtracted out the entire gene set for embryonic epidermal progenitors in *Xenopus* embryos, leaving 1742 transcripts that were upregulated in these Xenobots (Supplementary Data 2). ~41% (712 transcripts) of the transcripts were uncharacterized “LOC” genes. We used genome-wide functional annotation tool eggNOG-mapper^80^ to annotate these uncharacterized transcripts using human (most annotated genome) orthologs. We first tested the functional annotation using a small subset of already known *Xenopus* transcripts and found ~80% accuracy, with differences mostly being different versions of the same gene. After functional annotation of the uncharacterized transcripts, removing any remaining unannotated transcripts, and removing all duplicate transcripts from short and long chromosomes, we were left with 1078 uniquely upregulated transcripts in these Xenobots compared to age-matched *Xenopus* embryos (Supplementary Data 3).

We found many cilia and motor protein transcripts in the 1078 uniquely upregulated transcripts in Xenobots, which suggested the possibility that perhaps even after epidermal gene subtraction, there might still be some false positively enriched epidermal transcripts, particularly belonging to the epidermal multiciliate cells. To address this issue, we raised our stringency to very high (likely losing some true positives) by again resorting to Klein tools database of single-cell transcriptome of stage 22 entire Xenopus embryos. In addition to the epidermal progenitor gene subtraction already performed, we collected all the genes expressed in the different types of epidermal cells, namely, multiciliated cells, alpha and beta ionocytes, and goblet cells. We then subtracted any genes from any of these four cells that were present in our list of 1078 transcripts uniquely upregulated in these Xenobots. We then obtained a highly stringent list of 795 transcripts in which we had high confidence that these transcripts were uniquely upregulated in these Xenobots compared to age-matched embryos. After mapping these 795 transcripts to human orthologs, we obtained 537 transcripts, which were then used for subsequent comparisons and analysis (Supplementary Data 4).

### Functional analysis of transcripts uniquely upregulated in these Xenobots compared to age-matched Xenopus embryos

**Fig. 3:**
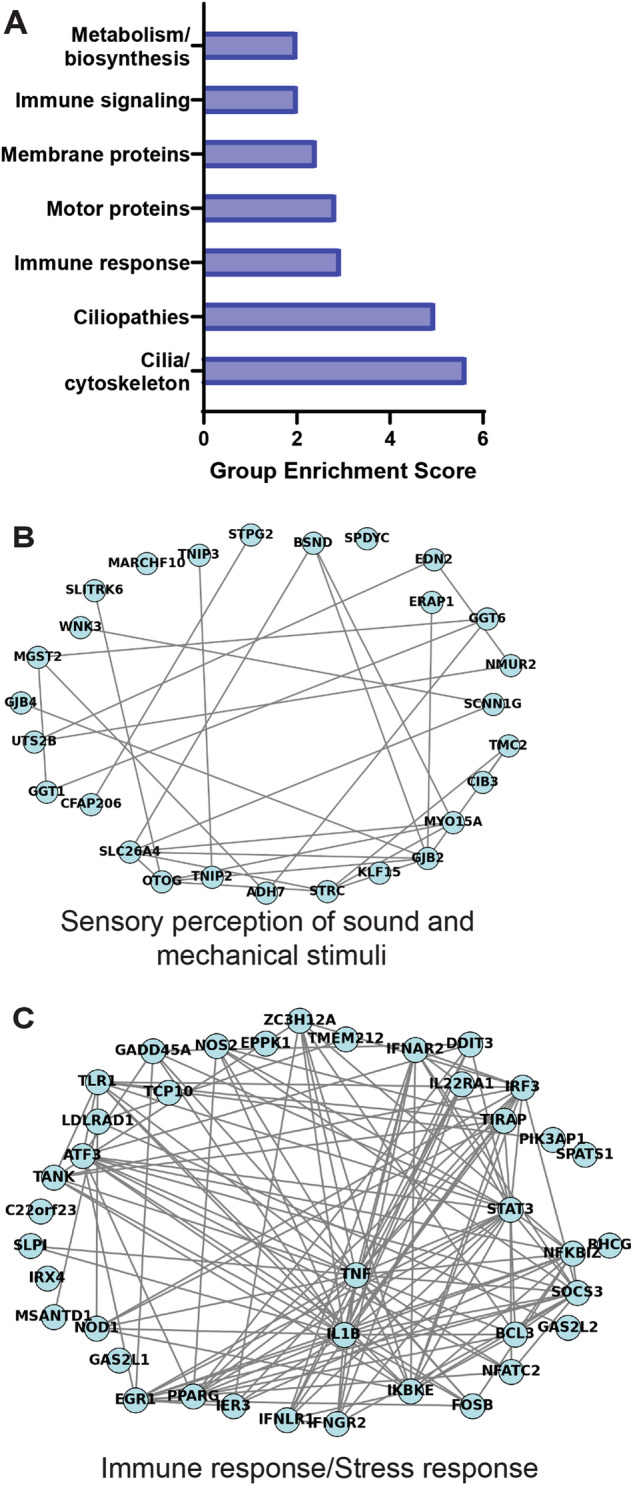
Functional enrichment and network clustering analysis of high stringency transcripts uniquely upregulated in Xenobots compared to age-matched *Xenopus* embryos. **A** Functional enrichment analysis showing enrichment of seven different biological categories. **B**–**C** Network clustering analysis identified 10 clusters (Supplementary Data 6), including cluster for sensory perception of sound and mechanical stimuli (**B**) and immune/stress response (**C**).

What kind of genes did the basal Xenobots upregulate? To understand the biological processes that might be affected by their unique transcripts, we first performed functional enrichment analysis using the Database for Annotation, Visualization and Integrated Discovery (DAVID)^81,82^. Using DAVID, we identified enriched biological themes amongst significantly differentially expressed genes and clustered the redundant annotation terms using the Functional Annotation Clustering tool. We identified the most enriched functional clusters and ranked them based on their group enrichment score, which is the geometric mean (in -log scale) of members’ *p* values in a corresponding annotation cluster (Fig. 3A and Supplementary Data 5). Interestingly, despite the very high stringency and removal of genes expressed by epidermal cells, we found enrichment of cilia, ciliopathies, and motor proteins. We also found categories for immune response and signaling, and metabolism/biosynthesis. Interestingly, we found a category of membrane proteins that contained largest number of genes belonging to various functions such as ion channels and transporters, gap junction and junctional proteins, calcium binding proteins, ATP binding proteins, sound or mechanical stimuli responsive proteins.

In addition, we also performed a network analysis to identify active functional biological modules^83,84^. We combined gene expression and interaction data and then applied network embedding, followed by clustering similarly to that performed by refs. ^85,86^. The detected functional modules were then enriched using g:profiler^87^. This network clustering analysis identified 10 enriched clusters (Supplementary Data 6). Many of the clusters were similar to the functional enrichment analysis (Supplementary Data 5 and 6), such as cilia/cytoskeleton/ciliopathies, immune and stress response, metabolism/biosynthesis (Fig. 3, Supplementary Fig. 3, and Supplementary Data 5 and 6). We also found clusters for extracellular matrix (ECM)/proliferation/multicellular organization, and sensory perception of sound and mechanical stimuli (Fig. 3, Supplementary Fig. 3, and Supplementary Data 6).

In summary, we identified biological processes enriched in these Xenobots compared to age-matched *Xenopus* embryos. These include cilia and motor proteins, tissue building/multicellular organization, immune and stress response, metabolism shift, and perception of sound and mechanical stimuli. Among these, the tissue building/multicellular organization and immune and stress response were unsurprising as these functions are required for building any novel morphology^88–92^. The presence of cilia and motor proteins in spite of our very high stringency, was surprising. Lastly, the shift in metabolism compared to embryo and the presence of sensory perception of sound and mechanical stimuli were extremely surprising.

### Transcripts uniquely upregulated in these Xenobots do not include any mesodermal, endodermal, or any axis patterning genes

To assess whether any mesodermal, endodermal, or axis patterning genes were being up-regulated in the basal Xenobots, we first curated a list of most prominent genes for each of these categories from literature (Supplementary Data 7). We then compared to see if any of these genes are expressed in our high-stringency transcripts upregulated in these Xenobots. None of the critical genes from mesoderm, endoderm, mesendoderm, anterior-posterior patterning, dorso-ventral patterning, or left-right patterning were found to be enriched in these Xenobots. This suggests that it is unlikely that these Xenobots perform transdifferentiation to recover their missing germ layers.

### Xenobots change their motion behavior in response to acoustic vibration stimuli

**Fig. 4:**
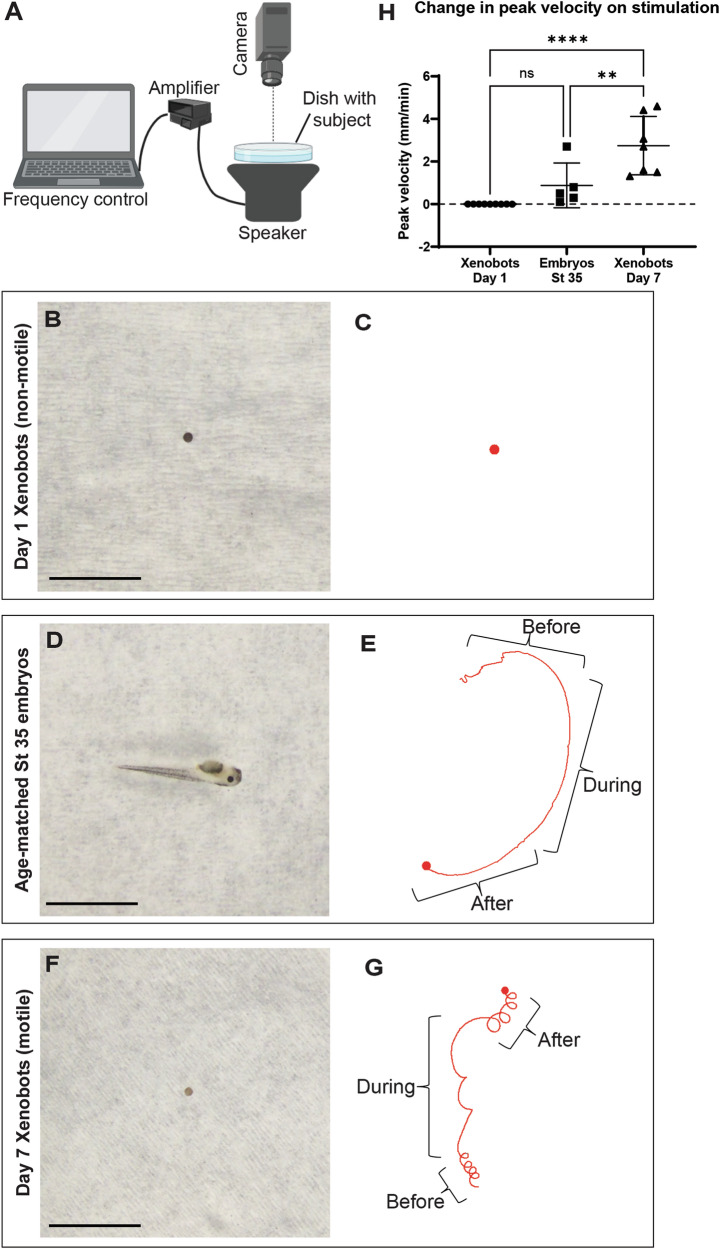
Xenobots respond to acoustic vibrations by changing motion behavior. **A** Experimental setup for exposure of subjects to 300 Hz acoustic vibration and time lapse recording of motion behavior for 10 min before exposure, 10 min during exposure, and 10 min after exposure. Created with BioRender.com. **B**–**H** Time lapse recording of motion behavior of day 1 non-ciliated, non-motile Xenobots, age-matched stage 35 *Xenopus* embryos, and day 7 autonomously motile Xenobots, along with motion tracking of their behavior and quantification of change in peak velocity from baseline during interval of 300 Hz vibration stimulus. **B**, **D**, **F** Representative image of day 1 non-motile Xenobots, age-matched stage 35 *Xenopus* embryos treated with tricaine to inhibit muscle movement while leaving cilia-based motion intact, and day 7 autonomously motile Xenobots, respectively. Scale bar = 5 mm. **C**, **E**, **G** Representative tracking of day 1 non-motile Xenobots, age-matched stage 35 embryos, and day 7 autonomously motile Xenobots, respectively, across the time intervals of before, during, and after 300 Hz vibration stimulus. **H** Quantification of change in peak velocity (millimeters/min) between the time intervals of before and during 300 Hz vibration stimulus. Data represented as mean ± SD. Xenobot Day 1—*n* = 9, Embryos St 35—*n* = 5, Xenobots Day 7—*n* = 7, ns-non-significant, **p < 0.01, ****p < 0.0001, repeated measures One-Way ANOVA with Tukey’s multiple comparison test.

Sensory perception of sound and mechanical stimuli was one of the most surprising gene clusters for biological processes identified to be significantly upregulated in Xenobots (Fig. 3). The possibility that basal Xenobots could exhibit novel functional responses to sound is fascinating as it would provide a tractable method to control Xenobot physiology and/or behavior, as well as shedding light on evolutionary developmental biology aspects of sensory capabilities. To functionally test this hypothesis, we exposed Xenobots to acoustic vibration stimulus and documented changes in their motion behavior (Fig. 4). First, we created an apparatus to apply acoustic vibration stimulus to Xenobots (Fig. 4A). A preliminary frequency survey showed that Xenobots respond to vibration stimulus by changing their motion behavior within the frequency range of 50 Hz–300 Hz with the strongest response at 300 Hz. Hence, we chose a frequency of 300 Hz for our experiments. The amplitude was reduced to the point where there was no visible movement of liquid in the dish, and then the frequency and amplitude were kept constant throughout all experiments. We recorded time-lapse videos of Xenobot motion for 10 min before the vibration stimulus, 10 min during the vibration stimulus, and for 10 min after the vibration stimulus (total 30 min of observation for each Xenobot). We first performed two essential controls.

To test whether subtle movement of liquid medium in response to vibration may be physically moving the Xenobot we used day 1 Xenobots as controls, because at that time point cellular differentiation has not yet occurred and there are no multiciliate cells: they are non-motile (Fig. 4B and Supplementary Movie 1), and thus would reveal any passive movement induced externally. Day 1 Xenobots are especially good controls as they have roughly the same size and mass as the day 7 autonomously motile Xenobots (Fig. 4F). Day 1 Xenobots showed no movement within the intervals before, during, and after vibration stimulus (*N* = 9, One-Way ANOVA with Tukey’s multiple comparisons test, *p* > 0.05) (Fig. 4C, H, and Supplementary Movies 1 and 2), suggesting that whatever movements we observe cannot be due to a passive displacement of inactive material by simple physical vibration.

Next, to test whether vibration stimulus causes a generic change in structure and/or function of cilia leading to change in motion behavior, we tested the effect of vibration stimulus on age-matched stage 35 embryos (Fig. 4D). In addition to cilia-based gliding, stage 35 embryos also have muscle-based movement. Hence, we treated them with 0.05% tricaine (MS-222)^93^ which blocks sensory-motor responses and thus inhibits muscle-based movement while leaving the cilia-based movement intact. Age-matched stage 35 embryos showed the expected cilia-based motion (Fig. 4E and Supplementary Movies 3 and 4) but did not show any significant difference in motion behavior between the time intervals of before, during, and after vibration stimulus (*N* = 5, One-Way ANOVA with Tukey’s multiple comparisons test, *p* > 0.05) (Fig. 4E, H, and Supplementary Movies 3 and 4). This result suggests that vibration stimulus is not causing a generic change in structure and/or function of cilia and shows no significant effect on cilia-based motion in age-matched stage 35 embryos. Would the Xenobots respond differently than embryos, as suggested by the RNAseq data?

We then tested the effect of vibration stimulus on day 7 autonomously motile Xenobots (Fig. 4F). Day 7 autonomously motile Xenobots show characteristic rotational motion behavior^33^ before exposure to vibration stimulus (Fig. 4G and Supplementary Movie 5 and 6). However, upon exposure to vibration stimulus, day 7 Xenobots show marked change in motion behavior with a more linear/arcing motion behavior and a significant increase in peak velocity (*N* = 7, One-Way ANOVA with Tukey’s multiple comparisons test, *p* < 0.01) (Fig. 4G, H, and Supplementary Movie 5 and 6). After the vibration stimulus they return to rotational motion behavior similar to pre-stimulus levels (Fig. 4G and Supplementary Movie 5 and 6). These results reveal that day 7 freely-behaving Xenobots specifically respond (in form of changed motion behavior) to vibration stimulus in ways that inactive (passive) Xenobots and age-matched normal embryos do not.

### Xenobot transcripts show enrichment of thanatotranscriptomic genes

We next turned our attention to additional aspects of the RNAseq results. Recent studies have shown the surprising fact that *cells* actively *turn on* specific genes after an organism’s death—the so-called thanatotranscriptome^71,73,94–97^. This is fascinating as it provides a way to dissociate death of an organism from that of its cells, thus shedding light on deep issues of multicellularity and emergent levels of organization in biology. We hypothesized that the data from human thanatotranscriptome studies indicated the cells’ attempt to survive in another form after the demise of the organism. In the case of mammals, living in dry air, this would be futile, but in the case of amphibians, this could work, and indeed, these Xenobots represent just that scenario—the original body is destroyed, but some of the cells live on in another form. Would these Xenobots express thanatotranscriptome genes?

As control we used all the genes expressed in *Xenopus* embryonic epidermal progenitor cells + multiciliated cells + alpha and beta ionocytes + goblet cells (obtained with Klein tools database), a total of 2635 genes (without LOC genes and mapped to human orthologs— Supplementary Data 8). From the literature, we curated the thanatotranscriptomic genes (336 genes) (Supplementary Data 9). Then we determined what percentage of transcripts uniquely upregulated in these Xenobots (537 genes mapped to human orthologs —Supplementary Data 4) are in the thanatotranscriptome set in comparison to the controls (total list of genes from all epidermal cell types—2635 genes—Supplementary Data 8). We found that the Xenobot-upregulated genes had more than double (3.5%) of thanatotranscriptome genes compared to control genes (1.6%) (Supplementary Data 10). Enrichment analysis of these Xenobot genes overlapping with the thanatotranscriptome suggests they mostly belong to immune activation, stress response, and insulin signaling. This suggests that the self-assembly process of these Xenobots following the harvesting of their source cells from an embryo may have similarities to the death process in other metazoan, including humans.

### Evolutionary age of enriched Xenobot transcripts

**Fig. 5:**
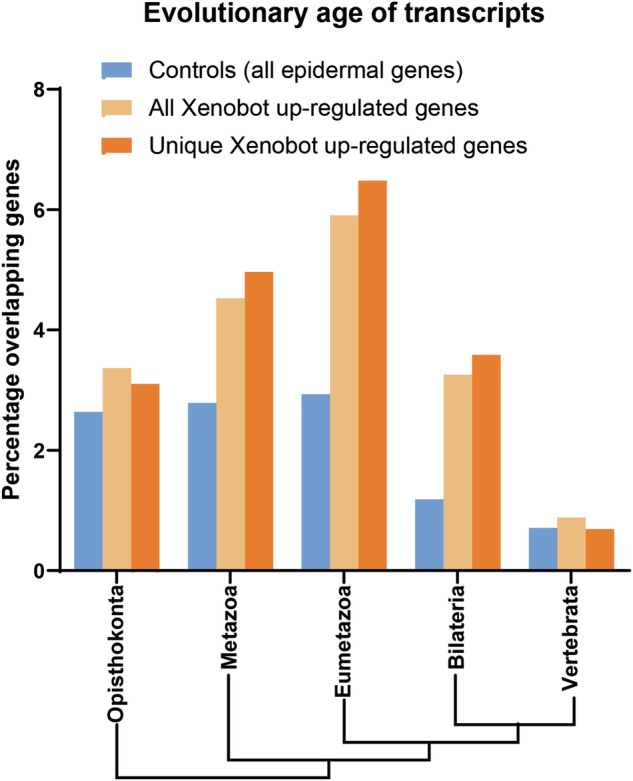
Xenobot transcripts are enriched in evolutionary older strata compared to control transcripts. Control transcripts are all the *Xenopus* genes expressed in epidermal progenitor cells + multiciliate cells + alpha and beta ionocyes + goblet cells (3374). Phylostratigraphic analysis of controls transcripts, all Xenobot upregulated transcripts (1812), and transcripts uniquely upregulated in Xenobots (1450), showing Xenobot transcripts significantly more enriched in Bilateria, Eumetazoa, and Metazoa compared to controls and the unique Xenobot upregulated transcripts more enriched than *all* Xenobot upregulated transcripts in these evolutionarily older strata. Chi-square test, *p* < 0.001.

To test the hypothesis that the basal Xenobots were acquiring more ancient transcriptomic patterns as a result of their morphogenesis and nascent emergent life history, we performed a phylostratigraphic analysis^98–103^. For this analysis, we did not need to remove LOC genes, and we did not need any mapping to human orthologs, so the fullest extent of the gene lists was used. We used PhylostratR, which applied BLAST to compare the whole *Xenopus* genome to at maximum 5 different proteomes of representative organisms of different ages in each evolutionary tree strata. Subsets of all genes in *Xenopus* genome associated with each stratum were shortlisted. Xenobot transcripts and control transcripts were then compared against these shortlisted genes. To determine the evolutionary age of transcripts from these Xenobots, we used both the unique Xenobot transcripts (1450 genes including LOC genes without mapping to human ortholog) and *all* the Xenobot up-regulated transcripts (1812 genes after removing short and long gene duplicates) and compared them to the controls (all the genes expressed in epidermal progenitor cells + multiciliated cells + alpha and beta ionocytes + goblet cells − 3374 genes including LOC genes without mapping to human orthologs). Going as far back as Vertebrata, there was not much difference between overlap of controls vs Xenobot transcripts. However, going further back in evolutionary time showed major enrichment of Xenobot transcripts overlap in comparison to controls for example, Bilateria, Eumetazoa, and Metazoa showed much more overlap with Xenobot transcripts than controls (Fig. 5). In addition, in these (Bilateria, Eumetazoa, and Metazoa) phyla the unique Xenobot genes showed more overlap than all Xenobot up-regulated genes (Fig. 5) indicating that the unique Xenobot genes were enriched for older phyla even in comparison to its own overexpressed genes suggesting specific enrichment of evolutionary older genes. Overall, this indicates that the transcripts upregulated in these Xenobots are particularly evolutionarily ancient genes.

## Discussion

In this study, we try to make inroads into the relationships between genomes, transcriptomes, and overall morphology^1,2^. This is a fundamental question in biology with many knowledge gaps concerning the range and plasticity of the latent space around a genome with respect to transcriptome and morphology, and lack of predictive power for the properties of novel constructs^20,104,105^ and chimeras^25,106^. Tremendous progress in the fields of synthetic biology, synthetic morphology, and biorobotics has made available biological model systems that are unencumbered by a history of environmental selection forces and serve as great model systems in which to understand the emergence of novel molecular-genetic, physiological, and morphological phenotypes.

In this study, we ask the question, how does the transcriptome change in response to cellular collectives acquiring non-evolved embodiments and behaviors in absence of any genomic editing, drug treatments, or heterologous interventions—to understand the top-down influence of morphology over transcriptome. Our goal was to utilize a synthetic living system to identify unique transcriptomic changes in it, in comparison to its native (wild-type) context. For this, we exploited the Xenobots platform, which includes multiple different kinds of autonomously-moving biobots all derived from *Xenopus* embryonic cells^32–35,37,107^. We analyzed the basal Xenobots, which are autonomously-motile, self-assembling multicellular entities derived from *Xenopus* ectodermal explants (animal caps)^33^. The *Xenopus* ectodermal explants have a rich history of serving as a tractable model system for investigating epidermal cell fate specification and cell fate plasticity in response to various chemical stimuli^49–55^. In addition, mucociliary organoids derived from them have helped understand mucociliary epidermis dynamics^56–61^.

Recent transcriptomic analysis has looked at epidermal cell fate specification during transformation of these ectodermal explants to the autonomously-moving entities (basal Xenobots)^56,62^. However, in this study, we investigate the transcriptome of these Xenobots in comparison to their native wild-type embryonic tissue using a different transcriptomic analysis beyond epidermal cell fate specification, to understand how these Xenobots’ morphology and behavior affect their transcriptomic changes. We found significantly greater inter-individual gene variability in these Xenobots, suggesting adaptive exploration of the transcriptional space to adapt to their novel embodiment and associated stressors. Gene ontology and network analysis of unique (non-epidermal marker) transcriptomic changes showed both expected shifts in multicellular organization and immune/stress response along with surprising changes in metabolism and sensory perception of sound and mechanical stimuli. Indeed, these autonomously motile basal Xenobots respond to acoustic stimulation by changing their motion behavior while motile age-matched embryos and passive non-motile Xenobots do not. Physlostratigraphic analysis showed transcriptional changes reflecting evolutionary ancient genes. We also found significant enrichment of thanatotranscriptomic genes, suggesting signatures of organismal death in them. Overall, all these transcriptomic changes in these Xenobots suggest that alteration of morphology and nascent emergent life history were enough to strongly affect the transcriptome (morphology/behavior driving gene expression).

Since these Xenobots are raised in different media concentration (0.75X MMR) as opposed to embryos (0.1X MMR), we first wanted to see if there are any major transcriptome changes in response to the different media concentration. Since the ectodermal explants were excised from embryos at stage 9 and incubated in 0.75X MMR for making these Xenobots, we incubated embryos in 0.75X MMR from stage 9 onwards until age-matched stage to mature day 7 Xenobots. Raising embryos in 0.75X MMR after stage 9 remarkably showed normal embryonic development with no developmental morphology defects, and embryos looking similar to their sibling raised in 0.1X MMR. Comparing transcriptome of embryos raised in 0.1X MMR to 0.75X MMR showed no major transcriptome differences (only 6 transcripts out of ~24,500 transcripts showed significant differences) (Supplementary Fig. 1). This was very surprising and suggests that the embryonic transcriptional regulation is very well shielded from external environmental changes. This might be due to the presence of tight junctions in the outmost ectodermal layer, which compartmentalizes the embryos from their external environment. If embryos are raised in 0.75X MMR from pre-stage 9, then they do not undergo normal gastrulation and do show developmental defects, suggesting as previously documented, that the pre-gastrulation embryos are much more susceptible to environmental differences than a post-gastrulation embryo.

To deal with false positives, we used scRNAseq database of Klein tools^79^. However, the caveat is that we had to use scRNA data from stage 22 embryos, whereas our age-matched embryos were stage 35. This stage 22 scRNA dataset is the best accessible results we have as there are no stage 35 scRNAseq datasets which we could use to remove epidermal progenitor transcripts. However, by stage 22, epidermal cell differentiation has already occurred^62,79^. In addition, to eliminate false positives, we took an extremely stringent approach by removing all genes expressed in epidermal progenitor cells, multiciliated cells, alpha and beta ionocytes, and goblet cells from our Xenobot dataset. In fact, this stringent curation may have resulted in the loss of some true positives, but it was worth it to give us good confidence in our results. Overall, our highly stringent list of Xenobot transcripts is a good starting point to look into unique transcriptional aspects of these Xenobots and to begin understanding how genome/transcriptome relates to novel morphologies. In future, single-cell RNA sequencing of Xenobots might allow us to look at regional and cell-type-specific expression patterns in Xenobots and how ectodermal progenitor cells distribution and profile diverge between Xenobots and embryos.

We compared and analyzed the transcriptome of these Xenobots with their age-matched *Xenopus* embryos to determine effects of altered morphology and nascent emergent life history on transcriptome. Our results give an indication of the kinds of transcriptional changes induced by change of morphology and behavior (no genomic editing or transgenes). We found at least 537 transcripts uniquely upregulated in these Xenobots (Supplementary Data 4). These transcripts indicated enriched functions such as locomotion apparatus assemblies (cilia-based motion and motor proteins), structure formation and multicellularity, stress and immune response, shift in metabolism, and sensory perception of sound and mechanical stimuli (Fig. 3, Supplementary Fig. 3, and Supplementary Data 5 and 6). The majority of these shifts were towards evolutionary older systems, which likely get suppressed when they are part of the organism. Phylostratigraphic analysis shows the transcripts uniquely upregulated in these Xenobots compared to the wild-type tissue are enriched for evolutionarily ancient genes and systems (Fig. 5). This is fascinating and suggests that cellular functional plasticity can roll backwards along the “ontogeny recapitulates phylogeny” axis, utilizing gene ensembles and systems from their distant evolutionary past that might be relevant in their new configuration and environment. Future work will examine possible links to similar phenomena (a shift toward ancient, unicellular transcripts) in cancer^108^ and potential implications for the relationship between cancer and multicellularity^109,110^.

The transcripts uniquely upregulated in these Xenobots compared to the wild-type tissue also showed enrichment for thanatotrascriptomic genes^71,73,94–97,111^, which are transcripts awakened in cells and organs after the overall organism dies. These transcripts mainly included immune/stress response and insulin signals. What this suggests is that these Xenobots have some sort of memory/information of them not being part of “living organism”. One possibility is that this might be evidence that the cells experienced the Xenobot construction event as the death of their parent organism; it is also likely that the thanatotranscriptome is limited by the fact they retained multicellularity; perhaps dissociating the cells and having them reboot multicellularity (as occurs in a different type of Xenobot^33^) might show a much stronger thanatotrascriptomic response. One challenge in this field has been that it was not possible to test whether these death-induced genes were functionally active. Indeed, what kind of function could even be assayed at the end of life? The Xenobots platform, as a whole, offers a path forward, because in future work, the thanatotranscriptome could be down-regulated via RNAi or morpholinos, to examine whether this would induce any negative effects on their continued survival or Xenobot functionality.

When cells are challenged and need to make quick changes to their gene expression to adapt to novel conditions, they exhibit exploratory adaptation by changing many different genes simultaneously, and different cells change different sets of genes to overcome the same challenge^66–69^. When comparing the overall transcriptome of the basal Xenobots and age-matched *Xenopus* embryos using PCA, the three Xenobots samples showed much wider separation compared to the three age-matched *Xenopus* embryo samples (Supplementary Fig. 2). Further, our analysis of gene count variability showed significantly higher inter-individual gene variability in these Xenobots compared to age-matched *Xenopus* embryos (Fig. 2). This increase in inter-individual gene variability in these Xenobots suggests that these Xenobots might be exploring the transcriptomic space (of possible gene expression profiles) to discover transcriptomes that allow them to improvise and adapt to their new embodiment. This is supported by the top ten most variable transcripts in these Xenobots which show prominent presence (three genes out of ten) of *oncomodulin* that encodes a protein usually found only during early embryonic cells and tumor cells and performs widely distinct functions^78^ including maintaining sensory perception, tissue regeneration, immune response, and strong antioxidant properties^78^. Other genes in the top ten most variable transcripts in these Xenobots include: *tenomodulin* which is involved in tissue maintenance by supporting stem cell renewal and preventing senescence and aging^112–114^, adhesion proteins *neurotrimin*^115^ and *salivary glue protein-3 like protein*^116^, *A. superbus venom factor 1* an immune regulator^117^, *somatostatin receptor 4* a regulator of hormone and secretory protein secretion^118^, *chloride intracellular channel 4* that belongs to an extremely conserved (across prokaryotes and eukaryotes) family that can perform different and independent functions at the membrane and the cytoplasm including fundamental cellular processes such as regulating mitochondrial function, exosome communication, membrane trafficking, and pH maintenance^119,120^, and *tyrosine aminotransferase* which is involved in gluconeogenesis— an alternate metabolic pathway^121^.

All of these functions are likely to be useful in the capacity to adjust to novel embodiments and challenges. Xenobots are an intriguing model in which to characterize problem-solving during traversal of transcriptional, physiological, and other unconventional spaces^88,122^. Individuals may explore the option space for many possible paths to adaptations to new ways of existence. Estimation of total cell number of a cohort of Xenobots reveals that they are remarkably similar in total cell number (Supplementary Fig. 4), ruling out large variability in cell numbers as a reason for transcriptomic differences (as also do prior studies showing embryos’ remarkable invariance to cell number^123,124^). Alternatively, the greater transcriptomic differences among these Xenobots may be a reflection of heterogeneity introduced to these Xenobot populations by transcriptional memory of the unique “lived experiences” of each Xenobot^125–127^. Finally, it has been observed that embryos in groups assist the morphogenesis of others and develop more uniformly with fewer defects^128^. While the embryo groups in the present study were small (10 individuals), they may still have some capacity to synchronize their transcriptomes that these Xenobots lack. In future studies, a spatial transcriptomic analysis of individual Xenobots would provide a better understating of this adaptive exploration process.

We saw not a single transcript overlap between the uniquely upregulated transcripts in these Xenobots in comparison to wild-type tissue and genes representing the mesoderm, endoderm, and axis patterning clusters (Supplementary Data 7), suggesting that there was no “contamination” of the explanted ectodermal tissue from which these Xenobots were derived with mesoderm, endoderm or axis patterning genes. Likewise, it did not support transdifferentiation of ectoderm cells into other lineages.

Overall, both functional enrichment analysis and the network clustering analysis showed largely overlapping functional clusters (Fig. 3, Supplementary Fig. 3, Supplementary Data 5, and 6). Even with the stringent subtraction conditions, including subtraction of all multiciliate cell genes, we still saw cilia assembly and motor proteins as major enrichment categories in both gene ontology analysis and network clustering analysis. This suggests that there might be significant upregulation of generation/making of multiciliate cells in these Xenobots compared to embryos. This is supported by our previous observation that Xenobots have >2 fold higher density of multicilated cells compared to age-matched embryos^33^. We did not observe much difference in cilia length and cilia number between Xenobots and age-matched embryos (Supplementary Fig. 5). In addition, the multiciliate cells in embryos may be much more stable and long-lasting compared to those Xenobots, which might be making them at a higher rate due to rapid turnover. This is partly supported by the observation of both proliferation and cell death categories being upregulated in these Xenobots, suggesting a higher turnover of cells. It is also possible that there are some unique and subtle differences in cilia assembly and adjoining motor proteins and their functions in these Xenobots’ morphology configuration. Overall, there is definitely a major shift in cilia assembly and motor proteins and perhaps multiciliate cell dynamics in these Xenobots; this remains an area of active investigation.

Subcategories involved in tissue building, repair, and multicellular organization were ATP binding, calcium binding, and subcategories in stress and immune response signals, mainly included cytokines, heat-shock proteins, and MMPs. It has been postulated that stress/immune response is important indicator of deviation from homeostasis and target morphology states and involved in moving the system by repair and tissue building processes to reach its final state^88–92^. It was interesting to see *V-ATPase* among these genes, as it is not only deeply conserved (evolutionarily ancient) but has also been postulated to be one of the key elements required for generation of multicellularity^129–131^, morphogenesis^132–135^, and cancer^136–138^. These genes represent candidates for control knobs that will be tested for their ability to enable guidance of the self-organization process to produce other types of Xenobots of different shapes and morphologies.

It was surprising to see sensory perception of sound and mechanical stimuli as an upregulated category in these Xenobots. Initially, we thought it might be erroneous or cross-assignment of cilia-related transcripts, but transcripts in this category were not cilia-related. They indeed contained transcripts known to be involved in sound and mechanical stimuli perception such as: *GJB2* which is a gap junction protein involved in sensory perception (among other functions) and mutational loss of this protein leads to hearing loss in humans and mice^139–141^, *stereocilin*^142^—the mechanoreceptor for sound waves/vibrations, proteins involved in inner ear membrane formation, inner ear ion channels, and many other stereocilia (inner ear hair cells) specific proteins. Importantly, the expression data were experimentally confirmed in a functional experiment. We tested the ability of these basal Xenobots to respond to acoustic vibration stimulus (Fig. 4 and Supplementary Movies 1–6). Observable changes in their motion behavior (Fig. 4 and Supplementary Movies 5 and 6) confirm that they do indeed respond to acoustic stimulus. This change in motion behavior is not due to physical movement of medium or simple mechanical effect of vibration on cilia function (Fig. 4 and Supplementary Movies 1–4), as indicated by controls (immotile bots and ciliated age-matched normal *Xenopus* embryos, neither of which responded to the stimulus).

Future work will detail the specific mechanisms leading from stimulus to change in behavior and characterize additional motility patterns that may be inducible by diverse stimuli. Importantly, the field of morphological computing reveals how the physical properties of bodies serve as an integral part of their cognition^143^. The Xenobots’ rich mix of ciliary action (itself a highly active multiscale system), internal bioelectric and calcium physiology, and fluid dynamics makes Xenobots an exciting model system in which to unravel morphological computing and behavioral responses in novel morphologies with wild-type genomes^144–149^.

Xenobots' behavioral response to acoustic stimulus suggests several points for consideration. First, these basal Xenobots express high levels of acoustic-related genes and are sensitive to such stimuli, whereas age-matched *Xenopus* embryos are not. The induction of whole cluster of transcriptomic machinery sufficient for a functional behavioral response, in a wild-type genome (in the absence of synthetic circuits or added induction by novel cell populations), may shed light on evolutionary developmental biology consequences of changes of morphology and nascent emergent life history. This in turn suggests a future research program to attempt to identify novel sensory-behavioral capabilities in other minimal, organoid, and bioengineered living constructs, which may heretofore have gone unnoticed. Future studies in organoid *behavior* and their potentially novel transcriptomics can offer new ways to exchange information with them, as shown here with Xenobots using acoustic stimuli identified through transcriptomics. Similar implications also apply to patient-derived-xenografts^150^. Second, from the perspective of biorobotics and the use of this model system as useful synthetic living machines, the ability to reversibly modulate their behavior via acoustic stimuli opens fascinating possibilities for control. Work is ongoing to map the frequency, waveform, and amplitude domains and link them to predictable changes in behavior (both via motility and via gene expression and physiological state). Real-time modulation of the hydroacoustic parameters in closed-loop controllers is a possible path forward for guided functionality in bio-robotics in Xenobots and beyond.

What would the role of such sensory perception of sound and mechanical stimuli be in such an aneural system like these Xenobots? And how does that regulate its morphology and movement behavior? A role of acoustics in aneural systems is not unprecedented. Recent studies have shown that coral larvae, which are aneural masses of cells with cilia-based movement similar to these Xenobots, perceive reef sounds (distinguishing healthy vs unhealthy reefs and mother reefs vs other healthy reefs), in order to migrate preferentially^151–154^. It is not known how (mechanism of action) they are able to achieve this acoustic perception, and it remains an area of investigation. Thus, it is possible that such acoustics-based sensing and movement is one of the evolutionarily ancient mechanisms of perceiving the environment and reacting to it via motion before nervous system evolved, leading to coordinated movement behavior. Given the presence of machinery for perception of sound and mechanical stimuli in these Xenobots, acoustic perception and response similar to coral larvae should be examined in Xenobots.

Although it is well known that yolk platelets provide the energy source and essential nutrients for *Xenopus* during embryonic development^155–157^, little is known about the metabolism happening within the embryos. Some evidence suggests that during early *Xenopus* embryonic development (pre-gastrulation), the pentose cycle and glutamate-aspartate cycle are predominant^158–160^. The Embden-Meyerhof pathway and Krebs cycle, although functional, contribute little during pre-gastrulation stages, but their relative contribution shows major increase after gastrulation^158–160^. Interestingly, in the basal Xenobots there seems to be upregulation of different metabolic processes, such as gluconeogenesis (which has been shown not to occur in embryos^159^) and ketone metabolism and metabolic process breaking down cholesterol and steroids. These were associated with presence of many liver-related transcripts involved in metabolism, although these Xenobots have no liver. It is interesting to see upregulation of gluconeogenesis and ketone metabolism in these Xenobots which are highly conserved metabolic processes of generating glucose from non-carbohydrate carbon substrates and is found in microorganisms, bacteria, fungi, plants, and animals^161–167^, suggesting that metabolically these Xenobots might be shifting to an ancient mechanism to meet their energy requirements. This is perhaps due to the majority of yolk (energy source of embryos) being on the vegetal side of the embryo, and since these Xenobots are derived from the animal side, they may have disproportionately lower yolk, thus needing to manage energy use given the limited amount of energy source. Interestingly, V-ATPase, the deeply conserved proton pump known to be involved in many functions (generating multicellularity, morphogenesis and cancer^129–138^) is also known to regulate mitochondrial metabolism^168,169^ and was found to be upregulated in these Xenobots as part of metabolism cluster, thus suggesting an overall shift in the metabolism in these Xenobots. Further detailed and deep investigation first into the metabolism and energy usage of embryos and then of these Xenobots will help reveal the shifts in metabolism. Understanding this will allow us to manage the energy requirements and control the overall nascent emergent life history of these Xenobots. Along these lines, it has been shown that external supply of glucose can drastically increase the life-span of these synthetic biobots^33^.

From the gene ontology and network analysis categories, it seemed that the basal Xenobots had activated evolutionarily ancient functions. To test the hypothesis that these Xenobots were acquiring more ancient transcriptomes as a result of their morphogenesis and nascent emergent life history, we performed a phylostratigraphic analysis. Here we compared the control transcripts (all transcripts from epidermal progenitor cells + alpha and beta ionocytes+, multiciliated cells + goblet cells) with transcripts uniquely upregulated in these Xenobots. The controls and Xenobot transcripts were equally enriched until Vertebrata strata. However, although the controls have a much larger transcript dataset (3374), the Xenobot transcripts (1450 uniquely upregulated transcripts) were much more enriched than controls in the older strata Bilateria, Eumetazoa, and Metazoa. This again suggests that these Xenobots have upregulated transcripts and programs that are evolutionarily ancient. Along those lines, the cellular physiology of these Xenobots appears to be closer to evolutionary ancient organisms with a single layer of differentiated cells with no nervous system and vasculature, and cilia-based movements. The phylostratigraphic analysis also shows that these Xenobot genes are evolutionarily basal and found in organisms with simpler body plan. This fits in line with the observation of shifts in metabolism, sensory perception process, and multicellular organization towards evolutionary ancient processes. This indicates that when cells and tissues are liberated from their organisms, they can tap into their evolutionary history to kickstart ancient processes and adjust their energy requirements to reach novel morphology goals.

Taken together, the results of this study reveal a control arc that is complementary to the conventional control of morphology by gene expression: to some extent, the genome is being used as a “resource book” by the cellular collectives to kick start various processes as per the conditions and needs of atypical multicellular configurations. Plasticity in form, function, and molecular physiology is gaining interest, as it impacts on basic evolutionary biology, biomedical applications, and synthetic bioengineering^89,170–176^, in way that augments current bottom-up approaches of DNA engineering and synthetic biology circuits. We see this work as part of a future roadmap in which a wide variety of synthetic systems are examined to see ways in which living organisms adapt to new environments on-the-fly and solve evolutionarily novel problems in transcriptional, morphological, and physiological spaces. Discovering how groups of cells manage their molecular-biological and metabolic resources to achieve adaptive ends in novel scenarios will facilitate the ability to control and generate different morphologies from the same genome by reshaping the morphological landscape to create new attractor states, control their behavior and responses, and manage their nascent life histories. Such knowledge will serve biomedical purposes (controlling multicellularity during regeneration, birth defects, and cancer), advance biorobotics and bio-AI (design systems with morphology and behavior to solve specific problems), and explore the space of possibilities of life-as-it-can-be.

## Materials and methods

### Animal husbandry

All experiments were approved by the University Institutional Animal Care and Use Committee (IACUC) under the protocol number M2023-18. We have complied with all relevant ethical regulations for animal use. *Xenopus Laevis* embryos were fertilized in vitro according to standard protocols^177^ and reared in either 0.1X Marc’s Modified Ringer’s solution or 0.75X Marc’s Modified Ringer’s solution (only post stage 9). *Xenopus* embryos were housed at 14 °C and staged according to Nieuwkoop and Faber^178^.

### Basal Xenobot construction

The basal Xenobots used in this study were derived from *Xenopus* embryonic ectodermal explants, also known as “animal caps”, as the starting material^49–52^. The basal Xenobots are the most basic version of Xenobots (no sculpting or engineering, or mixing of different tissues)^32^. These Xenobots were constructed manually from *Xenopus Laevis* embryos as described previously^33^. Briefly, in vitro fertilized embryos were reared at 14 °C in 0.1X Marc’s Modified Ringer’s solution (MMR) until Nieuwkoop and Faber stage 9^178^. At stage 9, embryos were transferred into a petri dish coated with 1% agarose made in 0.75X MMR and containing 0.75X MMR solution. Using surgical forceps, the vitelline membrane was removed from the embryos, and animal cap epidermal progenitor cells were cut out of the embryos as per previously described protocols^49–52^. The ectodermal explants were placed on the agarose with inside surface facing up. Over a period of 2 h the ectodermal explants round up into a spherical shape. These are then incubated at 14 °C on 1% agarose in 0.75X MMR for 7 days with daily cleaning. By day, 7 the tissue differentiates and transforms into an autonomously moving synthetic epidermal entity (basal Xenobot) used in this study. These Xenobots were not further modified and were used as mature at day 7.

### RNA extraction

Total RNA was extracted from either stage 35/36 *Xenopus Laevis* embryos or from autonomous moving, fully mature Xenobots (7 days post-extraction of ectodermal explants from embryos). For stage 35/36 embryos, we had 3 replicates for each experimental sample, and each sample contained 10 pooled embryos. For Xenobots we had 3 replicates for each experimental sample, and each sample contained 50 pooled Xenobots—necessary to gather enough RNA from markedly small mass of Xenobots (Fig. 2A). Total RNA was extracted using Tri-reagent (MRC, Inc.) as per the manufacturer’s protocol. Total RNA quality and quantity were measured using NanoDrop spectrophotometer (Thermo Fisher Scientific).

### rRNA depletion and RNA-sequencing

Total RNA was sent to the University Genomic Core. RNA quality was measured with bioanalyzer, and high-quality RNA was used for library preparation with the Illunima Stranded Total RNA with Ribo-Zero Plus. Libraries were multiplexed, and single-end 75-nucleotide sequencing was performed with 30 million reads per sample on Illumina NextSeq 550 High output. Raw read files were used for initial analysis at Bioinformatics and Biostatistics Core at Joslin Diabetes Center.

### Transcriptomic analysis

The reads were trimmed for adapter “CTGTCTCTTATACACATCTCCGAGCCCACGAGAC” and polyX tails, then filtered by sequencing Phred quality (≥Q15) using fastp^179^. *Xenopus laevis* genome sequences and gene annotation were downloaded from the NCBI genome database, version 10.1. GenomeGenerate module of the STAR aligner^180^ was used to generate the genome indexes. STAR aligner option was set to sjdbOverhang = 75 for 76-bp reads, as was ideal. Adapter-trimmed reads were aligned to the genome using STAR aligner with the two-pass option. Reads were mapped across the genome to identify novel splice junctions in the first-pass. These new annotations were then incorporated into the reference indexes, and reads were re-aligned with this new reference in the second pass. Gene expression was estimated from the gene alignments using RSEM tool for accurate quantification of gene and isoform expression from RNA-Seq data^181^. Low-expressing genes were filtered out by only keeping genes that had counts per million (CPM) more than 0.6 in at least 3 samples. There were 28,009 genes after filtering. Counts were normalized by weighted trimmed mean of M-values (TMM)^182^. Voom transformation^183^ was performed to transform counts into logCPM where logCPM = log2(106∗count/(library size∗normalization factor)). PCA analysis was performed to provide an overall view of the data. Differential gene expression analysis between groups was performed using limma^184^.

### Inter-individual gene count variability analysis

A schematic of the method is shown in Fig. 2A. The analysis was performed on gene count data from the 3 Xenobot pools and 3 age-matched *Xenopus* embryo pools in MATLAB. The 28,009 genes were ranked by the values of the means of their counts across all 6 pools of Xenobots and *Xenopus* embryos. Genes with a count of 0 in any of the 6 pools were removed, leaving 25,276 genes. Because the count value of a given gene in a given Xenobot pool represents the mean value of all the individual Xenobots in that pool, the standard deviation of the 3 Xenobot pools gives the standard error of the means (SE) of the Xenobots, and likewise for the age-matched *Xenopus* embryos. The SE is related to the number of individuals per pool (*n*) and the standard deviation of the individuals within the pools (*σ*) with the equation SE =  *σ*/sqrt(*n*). By multiplying the SE by sqrt(*n*) (where *n* = 50 for Xenobots, *n* = 10 for age-matched *Xenopus* embryos), *σ* can be calculated. Dividing *σ* by the mean count value of the 3 pools gives the coefficient of variation (CV) for Xenobots (CVX) and for age-matched *Xenopus* embryos (CVE). The CVs for all genes in the Xenobots and age-matched embryos were plotted as histograms with the area under the curves normalized to 1 (Fig. 2B) and the two distributions compared with a Wilcoxon Rank Sum test in MATLAB, returning *p* = 0, indicating highly significant difference between the distributions. The ranked gene list was then split into equal-size bins (percentiles), and the fraction of genes in each bin for which CVX > CVE was calculated and plotted in Fig. 2C, with black line indicating fraction of 0.5. The fraction of all genes for which CVX > CVE was found to be 0.9606 and was plotted as the red line. To assess whether any bin values were outside the range of values expected for random shuffles of the list, a permutation test was used to determine the typical distribution of bin values around the red line. First, the order of CVX–CVE gene pairs list was shuffled, and the bin analysis was performed on the shuffled list to determine a set of bin fractions. This was repeated 1000 times for different shuffles to produce a distribution of bin fraction values for each bin. To determine statistical significance of the true bin values’ differences from the mean (red line), each true bin fraction value was compared to the distribution. The *p* value was defined as the proportion of the distribution that was further (in absolute value) from the distribution mean (red line) than the true bin fraction. Bins with *p* values of *p* < 0.05 were deemed statistically significant and were colored dark blue; otherwise, bins were colored light blue, revealing significant variation of bin fractions across gene count percentiles. To generate the list of top 10 most variable Xenobot genes CVX values were used. For the list, genes for which any transcriptome in the group of 3 replicates had a count value of 0, and genes for which all count values in the 3 replicates were <10, were excluded.

### Functional enrichment analysis

To understand the biological processes and pathways enriched in RNA-seq comparisons, we performed functional enrichment analysis using the Database for Annotation, Visualization and Integrated Discovery (DAVID)^81,82^. Gene lists of interest (significantly differentially expressed genes with false-positive genes subtracted) were uploaded to the DAVID Analysis Wizard. Functional Annotation Clustering was performed to identify significantly enriched functional annotations, and these annotations were grouped into related clusters based on shared genes. Stringency classification was set to the default recommendation of “Medium”. Each individual annotation was assigned a *p* value, and each cluster of annotations received an enrichment score, which is the geometric mean (in −log scale) of members’ *p* values in a corresponding annotation cluster. Enriched functional clusters were ranked based on their group enrichment score.

### Xenobot network clustering analysis

We performed a network analysis for identifying active functional biological modules^83,84^. We combined gene expression and interaction data, extracted the xenobot protein-protein interaction, and applied network embedding followed by clustering, similarly to^85,86^. We used the MNMF network embedding and clustering algorithm^185^ and imposed a number of clusters equal to 15. To construct the synthetic proto-organism network, we filtered the genes of interest, found the human orthologs in the different conditions using HCOP^186^. Then, we extracted the corresponding network using the STRING database^187^. The detected functional modules after network embedding and clustering are enriched using g:profiler^87^.

### Phylostratigraphic analysis

We used phylostratR to perform the phylostratographic analysis of xenobot transcripts^188^. The focal species was set to “8355” for Xenopus Laevis. This package automates several key processes in evolutionary analysis (1) it constructs a clade tree from species listed in UniProt, aligned with the latest NCBI tree of life; (2) it prunes the clade tree such that it retains a phylogenetically diverse selection of representatives for each phylostratum; (3) it compiles a database of protein sequences from hundreds of species from the constructed clade tree and sourced from the UniProt Proteome database (in total 329 species in our study, including the yeast and human proteomes that we added manually) (4) it applies similarity search by conducting pairwise BLAST between the proteins encoded in the focal species (here the Xenopus Laevis) and all proteins from all target species within the clade tree; (5) it determines the “best hits” and infers homology for each gene of the focal species against each target species; (6) it assigns each gene to a phylostratum correlating with the oldest clade for which there is an inferred homolog. Genes found exclusively in the focal species are identified as orphan genes and are categorized under the phylostratum “Xenopus Laevis”. The phylostratas we selected are: All living organisms (Eubacteria, bacteria and their descendants), Eukaryota, Opisthokonta, Metazoa, Eumetazoa, Bilateria, Deuterostomia, Chordata, Vertebrata, Gnathostomata, Euteleostomi, Sarcopterygii, Tetrapoda, Anura, Xenopus, Xenopus Laevis. We then mapped the age of genes with our xenobot overexpressed genes in the different conditions and the control.

### Mapping to human orthologs

For the network analysis and comparisons with the thanatotranscritome, we mapped *Xenopus* genes to their human orthologs using HCOP^189^.

### Acoustic vibration stimulus setup

For controlling and providing acoustic vibration stimulus, we used a setup (Fig. 4A) containing a speaker (RECOIL RW8D2 Echo Series 2-ohms, 400 Watts) connected to a digital audio amplifier (Kinter K3118 Texas Instruments, 20 Watts output) operated using a laptop computer. An online tone generator (https://www.szynalski.com/tone-generator/) was used to set a sine-type wave with frequency of 300 Hz. The computer volume and online tone generator volumes were set to 100%. A P60 petridish coated at the bottom with 2–3 mm layer of 1% agarose made in 0.75X MMR was placed on top of the speaker. Fourteen milliliters of 0.75X MMR was added into the petridish. The volume of amplifier was set to 3.5 at which point no physical movement of media in the dish was observed in response to acoustic vibration stimulus. These settings were kept unchanged throughout all acoustic vibration stimulus experiments. An iPod camera mounted on a Stemi SV6 dissection microscope eyepiece was used to capture time-lapse imaging. Subjects (day 1 Xenobots, day 7 Xenobots, or age-matched stage 35 embryos) were placed in the center of petridish. Time-lapse recording was conducted for 10 min before initiation of vibration stimulus, followed by 10 min of vibration stimulus, and lastly 10 min after vibration stimulus was tuned off.

### Behavior imaging and analysis

Movement behavior was recorded using an iPod camera mounted on a Stemi SV6 dissection microscope eyepiece. ProShot app was used to capture time-lapse imaging of one frame every 2 s. Time-lapse recordings were compressed to 30 frames per second movie which results in 30 s movies of 30 min of motion tracking (10 min before stimulation, 10 min during stimulation, and 10 min after stimulation). Thus 1 s of movie = 1 min of actual time. Motion tracking of behavior captured in time-lapse videos was done using Ethovision XT v.15 (Noldus Information Technology). A scaled background image was used to set scale for distance. Motion tracking was completed and recorded. Any tracking errors were manually corrected in the track editor. All *X*–*Y* coordinates of each subject for every frame across the time-lapse video were exported for data analysis.

### Estimation of total cells in Xenobots

Xenobots were manufactured from embryos obtained from multiple female frogs. On maturity (with autonomous movement) to day 7, Xenobots were fixed in MEMFA fixative (100 mM Mops pH 7.4, 2 mM EGTA, 1 mM MgSO_4_, and 3.7% (v/v) formaldehyde) for 4 hours at room temperature on a nutator, followed by 3 washes for 15 min each with PBST (phosphate-buffered saline + 0.1% Tween 20) and stored at 4 °C until ready for staining. Fixed Xenobots were first imaged using Nikon SMZ-1500 microscope at 6X with a Teledyne Infinity camera and Infinity Analyze software, and two axial radius’ a and b were measured. Xenobots were then cut through the middle into two halves and stained with nuclear stain NucBlue (Thermofisher R37605—2 drops/ml) for 30 min followed by 3 washes for 5 min with PBST. The interior cut surface of the Xenobot was imaged for detecting nuclei using Leica Stellaris Sp8 confocal microscope with 16X water objective and DAPI excitation and emission settings. A 7 µM depth was imaged. Using the image the third axial radius c of Xenobot was measured. Using Fiji/imageJ total number of nuclei in the Xenobot section was counted, followed by calculating the area of that Xenobot section, volume of the imaged Xenobot section (area X depth—7 µM), nuclei per unit volume (total number of nuclei/calculated section volume), total volume of that Xenobot (Total volume = (4/3) * (22/7) * radius-a * radius-b * radius-c), and finally total nuclei in that Xenobot (Nuclei per unit volume * Total volume).

### Cilia immunostaining and quantification

Cilia were visualized by immunostaining with monoclonal anti-acetylated α-tubulin antibody (Sigma-Aldrich, T7451) using previously described protocol^190^. *Xenopus* embryos were obtained from multiple female frogs. A subset of the embryos was used to manufacture Xenobots, and rest were used as age-matched control embryos. On maturity (autonomous movement in Xenobots) at day 7, Xenobots and age-matched embryos were fixed in MEMFA fixative (100 mM Mops pH 7.4, 2 mM EGTA, 1 mM MgSO_4_, and 3.7% (v/v) formaldehyde) for 4 h at room temperature on a nutator, followed by 3 washes for 15 min each with PBST (phosphate-buffered saline + 0.1% Tween 20) and stored at 4 °C until ready for processing. Samples were first blocked with 10% goat serum in PBST at room temperature for 1 h. Samples were then incubated in monoclonal anti-acetylated α-tubulin antibody diluted at 1:1000 in the block (10% goat serum in PBST) at 4 °C overnight on a rocker. Samples were then washed 3 times for 15 min each in PBST followed by incubation in Alexa Fluor 555-conjugated goat anti-mouse secondary antibody at 1:500 dilution at 4 °C overnight. Finally, samples were washed 3 times for 15 min each in PBST. Cilia were imaged using Leica Stellaris Sp8 confocal microscope, 40X water objective with 4X zoom and Alexa-555 excitation and emission settings. Cilia length numbers were counted using Fiji/ImageJ software. Number of cilia per multiciliated cell and cilia length were counted from multiple multiciliated cells from each sample and more than 4 different samples in each treatment group.

### Statistics and reproducibility

Statistical analyses and visualizations were conducted using software appropriate for that experimental setup. The statistical methods, tests, and software employed for each experiment are varied and explained in detail in the methods, text, and figure legends, wherever applicable. Reproducibility was determined by conducting all experiments with a minimum of three independent biological replicates representing independent samples derived from independent batches. Details for each experiment are outlined in respective methods, text, and figure legends.

### Reporting summary

Further information on research design is available in the Nature Portfolio Reporting Summary linked to this article.

## Supplementary information

Supplementary Figs.

Description of Additional Supplementary Files

Supplementary Data 1

Supplementary Data 2

Supplementary Data 3

Supplementary Data 4

Supplementary Data 5

Supplementary Data 6

Supplementary Data 7

Supplementary Data 8

Supplementary Data 9

Supplementary Data 10

Supplementary Movie 1

Supplementary Movie 2

Supplementary Movie 3

Supplementary Movie 4

Supplementary Movie 5

Supplementary Movie 6

Reporting Summary

## Data Availability

RNA-sequencing data generated during the study are available in the NCBI GEO public repository with accession numbers (GSE275807 and GSE277182). Curated data are available in the Supplementary Data. Any other data are available from the corresponding author upon request.
