# Supplementary material for: Basal Xenobot transcriptomics reveals changes and novel control modality in cells freed from organismal influence
Source: Commun Biol. 2025 Apr 22;8:646. doi: 10.1038/s42003-025-08086-9 (PMC12015265; doi:10.1038/s42003-025-08086-9)
Supplement: Supplementary file 1 — Supplementary Figs. [file 42003_2025_8086_MOESM1_ESM.pdf]

Basal Xenobot Transcriptomics: Analysis of Gene Expression Changes in wild-type cells freed from the influence of the rest of the organism reveals novel control modality

Vaibhav P. Pai[1], Léo Pio-Lopez[1], Megan M. Sperry[1,2], Patrick Erickson[1], Parande Tayyebi[1], and Michael Levin[1,2*]

[1] Allen Discovery Center at Tufts University, Medford, MA, USA

[2] Wyss Institute for Biologically Inspired Engineering, Harvard University, Boston, MA, 02115, USA

* Corresponding Author:
Dr. Michael Levin
Allen Discovery Center at Tufts University,
Center for Regenerative and Developmental Biology,
Tufts University
200 Boston Avenue, suite 4600
Medford, MA 02155-4243
Phone: +1 617 627 6161
Email: michael.levin@tufts.edu

Running title: Unique Xenobot transcriptome

Keywords: Biological robots, synthetic living machines, transcriptome, RNAseq, vibration, sound, auditory

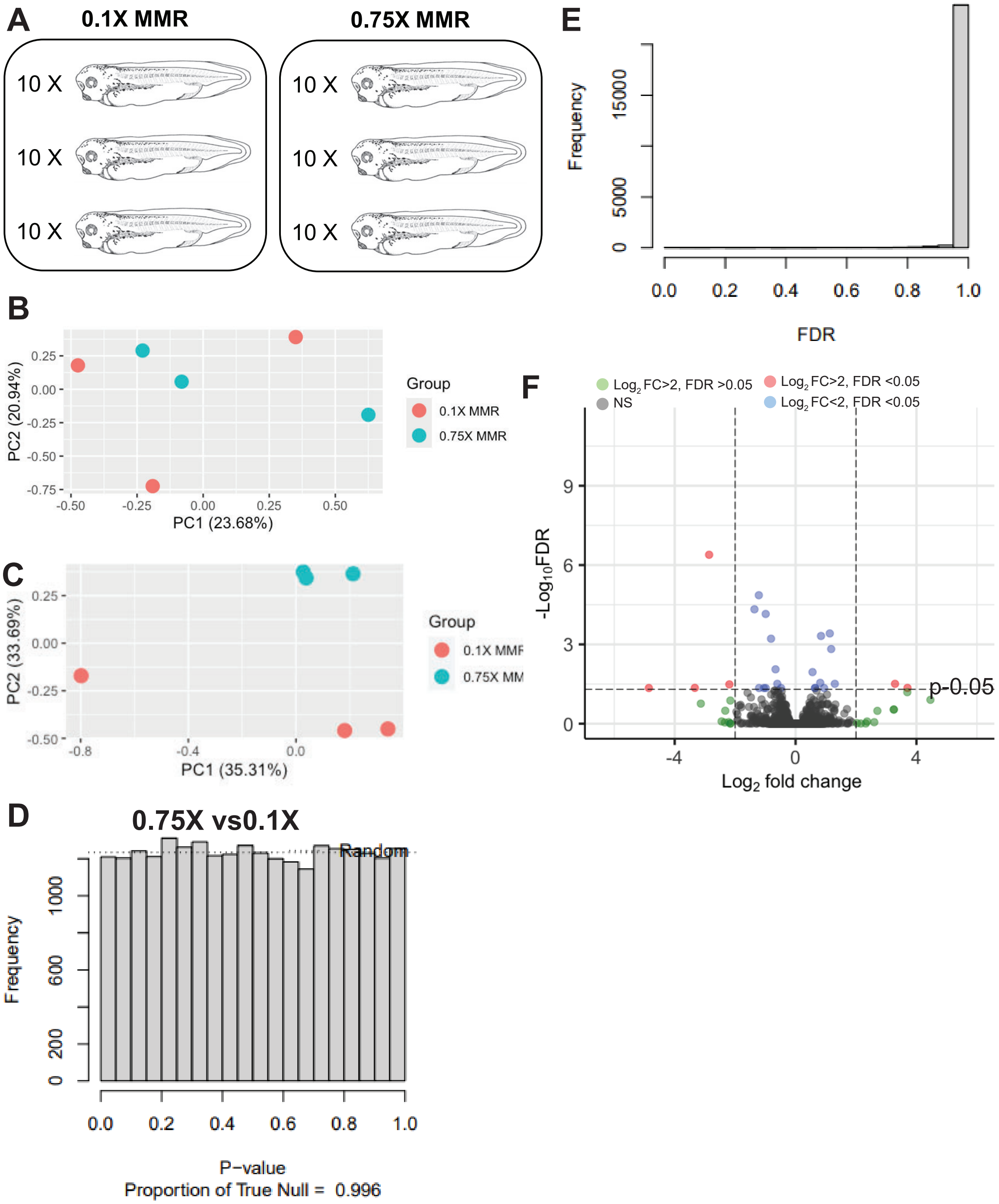

Supplementary Figure 1: No major transcriptional changes in stage 35/36 *Xenopus* embryos raised in 0.1X MMR and 0.75X MMR. (**A**) Schematic of the experimental setup with three replicates for each 0.1X MMR and 0.75X MMR rearing condition and each replicate having ten stage 35/36 embryos. (**B**) Principal component analysis plot of RNA-sequencing data from stage 35/36 *Xenopus* embryos raised in 0.1X MMR and 0.75X MMR showing no clustering. (**C**) Principal component analysis plot using surrogate variable adjusted data. (**D**) Histogram of *p*-value significance. If no genes are associated with phenotype the *p*-value histogram is expected to be relatively flat. Also, proportion of true null hypothesis (non-significant genes) is shown (**E**) Histogram of FDR significance. If no genes are associated with phenotype all the FDRs will be near one. (**F**) Volcano plot for differential expression between embryos raised in 0.1X MMR and 0.75X MMR. Significantly changed genes are highlighted in red.

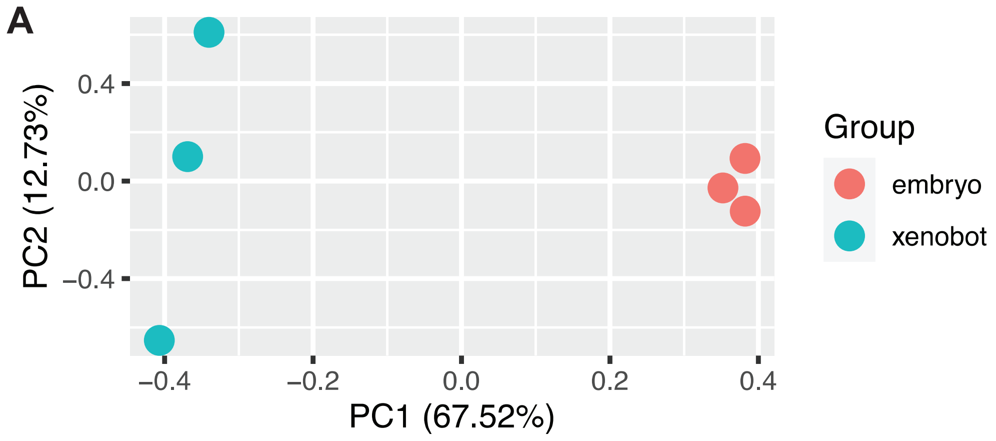

Supplementary Figure 2: Principal component analysis plot of RNA-sequencing data using surrogate variable adjusted data for Xenobots and age-matched stage 35/36 *Xenopus* embryos showing distinct separation of the two groups.

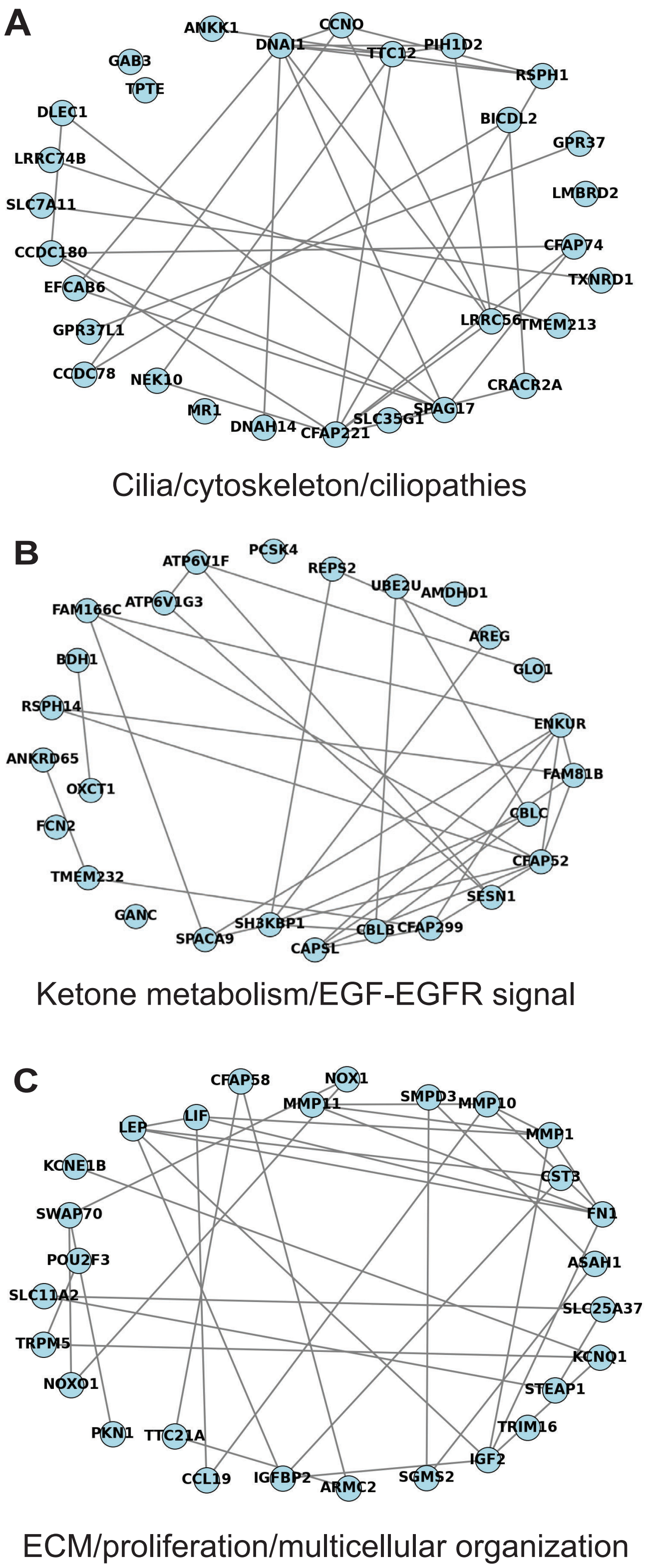

Supplementary Figure 3: Network clustering analysis of high stringency transcripts uniquely upregulated in Xenobots compared to age-matched *Xenopus* embryos. (**A-C**) Network clustering analysis identified 10 clusters (Supplemental Dataset 6) including clusters for cilia and cytoskeleton (**A**), Ketone metabolism and EGF/EGFR signal (**B**), and ECM/proliferation/multicellular organization (**C**).

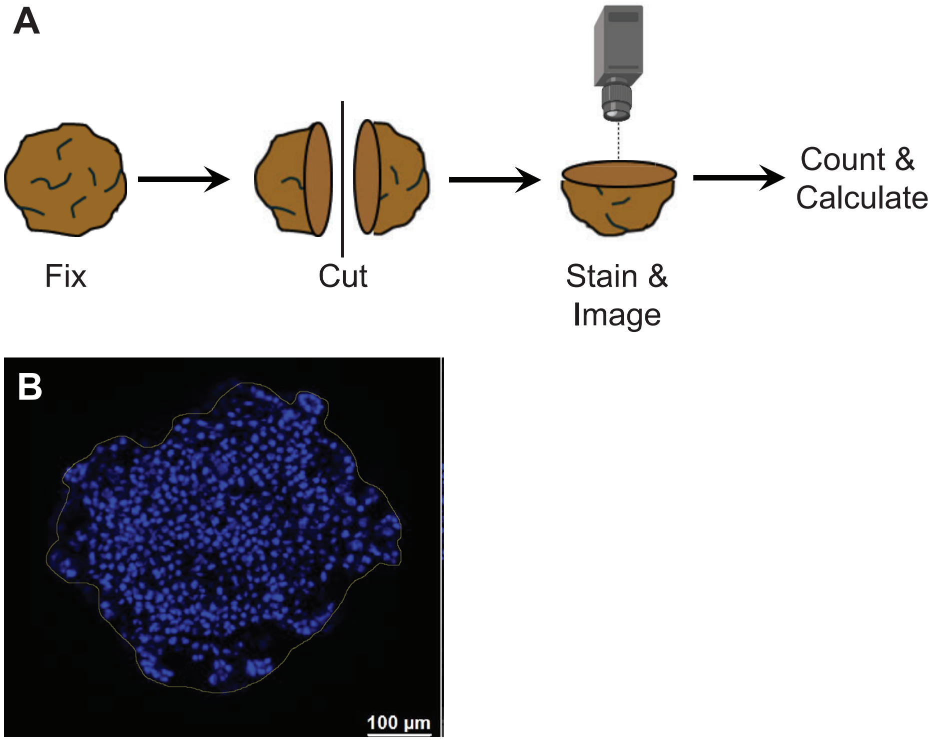

C

Estimated total cell count in Xenobots				
Xenobot #	Actual nuclei per unit volume (per µM^3)	Estimated Total nuclei	Average	Standard Deviation
Xenobot 1	$5.9X10^{-4}$	37736.64		
Xenobot 2	$6.8X10^{-4}$	36571.51		
Xenobot 3	$6.7X10^{-4}$	39000.62	38402.87	1223.62
Xenobot 4	$5.7X10^{-4}$	38376.03		
Xenobot 5	$5.2X10^{-4}$	40224.82		
Xenobot 6	$5.2X10^{-4}$	38507.58		

Supplementary Figure 4: Xenobots have relatively similar total cell numbers. (**A**) Schematic of the Xenobot total cell count pipeline. Xenobots were fixed, their axial dimensions measured, cut through the middle into two halves, stained with nuclear stain followed by imaging nuclei in the interior, counting and calculation to obtain total nuclei per Xenobot. (B) Representative image of stained nuclei in the interior of Xenobot. (**C**) Table showing actual nuclei per unit volume imaged and estimated total cell count in Xenobots.

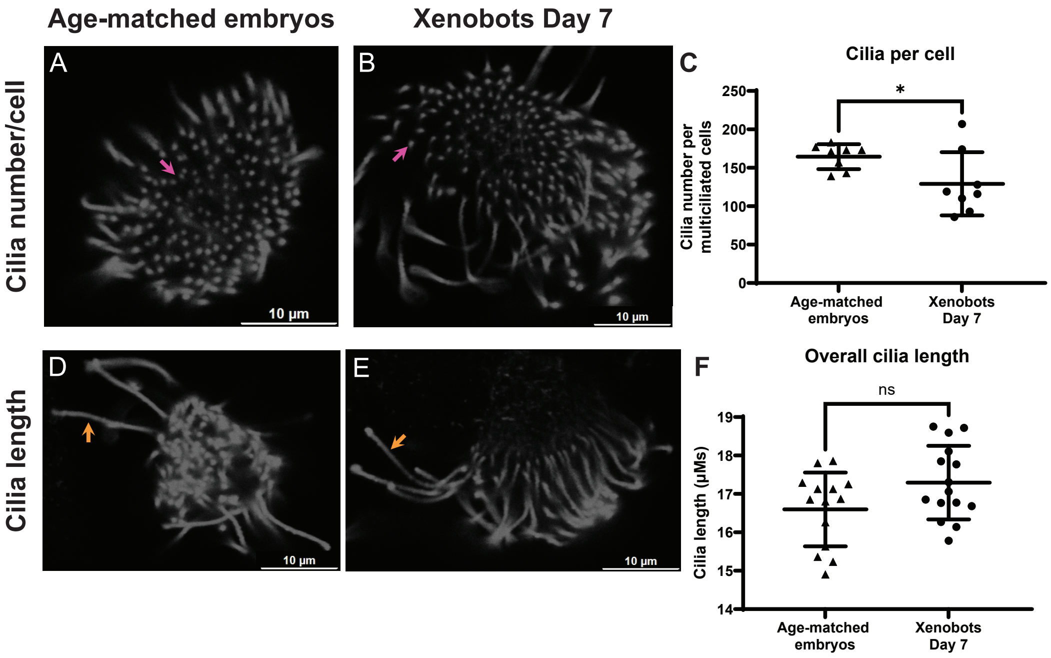

Supplementary Figure 5: Xenobots have overlapping cilia characteristics with age-matched embryos. Immunostaining for cilia in Xenobots and age-matched embryos. (**A & B**) Representative images at the base of cilia of multiciliated cells showing each cilium as a punctate spot (magenta arrows). (**C**) Quantification of number of cilia per multiciliated cell n=8, *-p=0.04, unpaired t-test. (**D & E**) Representative images of cilia length in multicilated cells (orange arrows). (**F**) Quantification of cilia length n>13, ns-non-significant, unpaired t-test. Data represented as mean $\pm$ SD.
