# Supplementary material for: Basal Xenobot transcriptomics reveals changes and novel control modality in cells freed from organismal influence
Source: Commun Biol. 2025 Apr 22;8:646. doi: 10.1038/s42003-025-08086-9 (PMC12015265; doi:10.1038/s42003-025-08086-9)
Supplement: Supplementary file 2 — Description of Additional Supplementary Files [file 42003_2025_8086_MOESM2_ESM.docx]

Description of Additional Supplementary Files

**File name:** Supplementary Data 1

**Description:** List of genes significantly changed in stage 35/36 embryos raised in 0.75X MMR in comparison to those raised in 0.1X MMR.

**File name:** Supplementary Data 2

**Description:** List of genes significantly upregulated in Xenobots compared to age-matched Xenopus embryos after subtracting epidermal genes.

**File name:** Supplementary Data 3

**Description:** List of genes significantly upregulated in Xenobots compared to age-matched Xenopus embryos after uncharacterized transcript annotation and duplicate removal.

**File name:** Supplementary Data 4

**Description:** List of genes significantly upregulated in Xenobots compared to age-matched embryos after subtracting any genes expressed in the epidermal cell types; multiciliate cells, alpha and beta ionocytes, and goblet cells.

**File name:** Supplementary Data 5

**Description:** Functional enrichment categories and their enrichment scores for high stringency transcripts uniquely upregulated in Xenobots compared to age-matched Xenopus embryos.

**File name:** Supplementary Data 6

**Description:** Network clustering analysis clusters enriched in high stringency transcripts uniquely upregulated in Xenobots compared to age-matched Xenopus embryos.

**File name:** Supplementary Data 7

**Description:** List of critical genes in mesodermal, endodermal, and axis patterning.

**File name:** Supplementary Data 8

**Description:** List of genes encompassing epidermal progenitors, alpha and beta ionocytes, multiciliated cell, and goblet cells that was used as controls for comparisons.

**File name:** Supplementary Data 9

**Description:** Curated list of thanatotranscriptomic genes.

**File name:** Supplementary Data 10

**Description:** List of genes upregulated in Xenobots that are part of the thanatotranscriptome.

**File name:** Supplementary Movie 1

**Description:** Time-lapse recording of representative day 1 Xenobot before, during, and after acoustic vibration stimulation.

**File name:** Supplementary Movie 2

**Description:** Track recording of motion of representative day 1 Xenobot before, during, and after acoustic vibration stimulation.

**File name:** Supplementary Movie 3

**Description:** Time-lapse recording of representative age-matched stage 35 Xenopus embryo before, during, and after acoustic vibration stimulation.

**File name:** Supplementary Movie 4

**Description:** Track recording of motion of representative age-matched stage 35 Xenopus embryo before, during, and after acoustic vibration stimulation.

**File name:** Supplementary Movie 5

**Description:** Time-lapse recording of representative day 7 Xenobot before, during, and after acoustic vibration stimulation.

**File name:** Supplementary Movie 6

**Description:** Track recording of motion of representative day 7 Xenobot before, during, and after acoustic vibration stimulation.

**File name:** Supplementary Data

**Description:**
